# Sarcoidosis and spondyloarthritis: A coincidence or common etiopathogenesis?

**DOI:** 10.22088/cjim.9.1.100

**Published:** 2018

**Authors:** Hamdi Wafa, Miladi Saoussen, Kaffel Dhia, Zouch Imen, Kchir Mohamed Montacer

**Affiliations:** 1 Department of Rheumatology, Kassab Institute of Orthopedics, Manouba. Tunisia.

**Keywords:** Sarcoidosis, Ankylosing Spondylarthritis, Tumor Necrosis Factor Alpha

## Abstract

**Background:**

Sarcoidosis is a multisystem granulomatous disease. Co-existence with spondyloarthritis (SA) has been more described as an adverse effect of anti-TNF α therapy than an association. We report herein a case of a typical sarcoidosis confirmed by histological proofs and an advanced SA with a bamboo column.

**Case Presentation:**

A 48-years-old woman presented with inflammatory back pain for 5 years and ankle swelling for 1 year. On physical examination, she had an exaggerated dorsal kyphosis and disappearance of lumbar lordosis with limitation in motion of the cervical and lumbar spine. Laboratory tests did not show an inflammatory syndrome or hypercalcemia. Plain radiographies of the spine and pelvic revealed a triple ray appearance with sacroiliitis grade 4. Chest radiography and CT confirmed the presence of bilateral hilar lymph nodes and parenchymal nodes. Bronchoscopy and biopsies were performed showing non-calcified granulomatous reaction without cell necrosis. The diagnosis of SA was performed based on 9 points of Amor criteria associated with pulmonary sarcoidosis. She was treated with 15 mg per week of methotrexate and 1mg/kg/day of prednisone for pulmonary disease with good outcomes.

**Conclusions:**

Sarcoidosis may be associated to SA besides paradoxical drug effect. The same physio pathological pathways mediate by TNF α are arguments for association than hazardous coincidence.

Sarcoidosis is a multisystem disease characterized by the occurrence of a non-calcified granulomatous reaction. The exact pathogenesis of this disease is still unknown, but it seems that it is caused by an inappropriate cellular immune response against environmental or infectious agents (1). This disease may present with different clinical features. It can mimic several rheumatic diseases or can develop concomitantly to them. The sacroiliac joint involvement is less frequent and can be found in 6.6% cases among patients with authentic sarcoidosis (2). Sacroiliitis is the hallmark of spondyloarthritis (SA). SA encompasses several chronic rheumatic disorders that share clinical features and a genetic association with human leukocyte antigen B27 (HLA-B27) (3). Common symptoms of SA-related diseases include inflammatory back pain, peripheral arthritis, enthesitis, and extra-articular features, such as psoriasis, inflammatory bowel disease, and uveitis. Most cases of co-existing sarcoidosis and SA published are described as an adverse effect of anti-tumor necrosis factor alpha (TNF α) therapy (4). Since both diseases share a same pathophysiological background based on the role of TNF α, co-existence seems to be an association rather than a hazardous event. We report herein a new case of pulmonary sarcoidosis discovered simultaneously associated to an axial evolved SA naïve of anti-TNF α therapy. 

## Case Presentation

**Figure 1:**
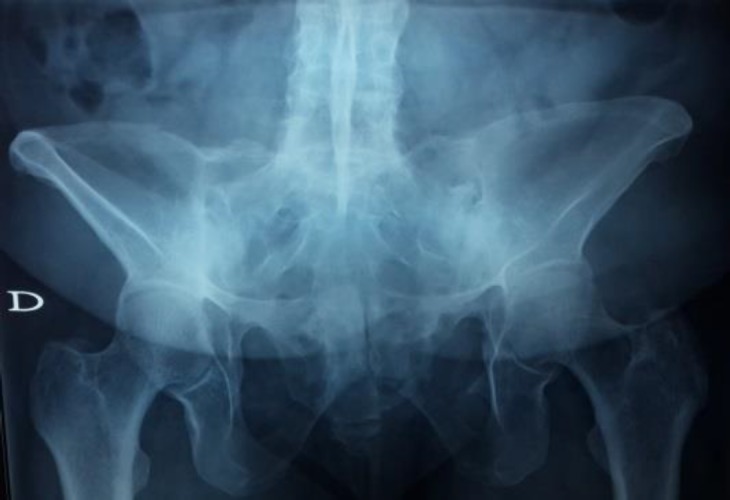
Pelvic x-rays showed a bilateral sacroiiitis grade 4

**Figure 2:**
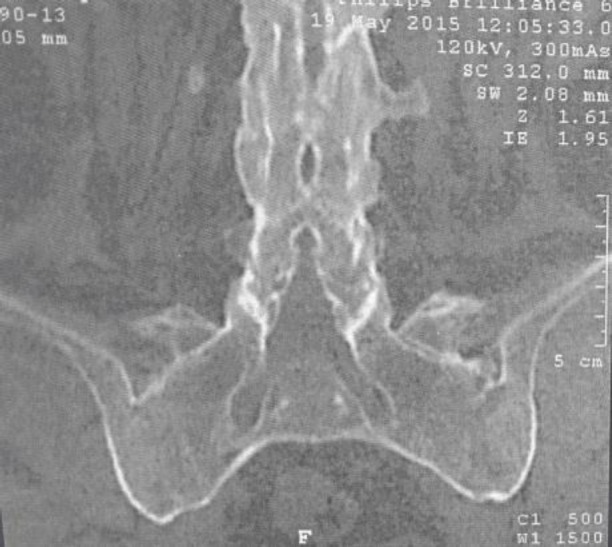
Frontal view of the sacroiliac joints showing sarcoiliitis grade 4 of Forestier

A 48 years-old woman was admitted in January 2015 to the Rheumatology Department of the National Institute of Orthopedics of Tunisia with complaints of inflammatory low back pain and a morning stiffness of one hour for 5 years. The history of the patient has revealed a recent swelling and heel pain and red eyes two times last year. She declared no shortness of breath or other breathing troubles. Furthermore, the patient’s personal and family past medical history was unremarkable. On physical examination, she had typical SA appearance with exaggerated dorsal kyphosis and disappearance of lumbar lordosis. A significant limitation in motion of the cervical and lumbar spine was noted and the Faber’s test and Patrick’s test were positive. Peripheral joint examination revealed swelling of ankles. Tenderness was noted in the insertion of Achilles tendon and plantar fascia. Systemic evaluation was normal except the crackle sounds in the lower zones of lungs. A routine laboratory assessment was unremarkable with a normal rate of serum calcium, sedimentation rate and C-reactive protein. Tuberculin skin test was negative. X-ray examination revealed typical finding of SA with a bilateral sacroiliitis grade 4 (figure 1), a triple ray in the lumbar and cervical spine. A pelvic CT confirmed the sacroiliitis fusion (figure 2).

**Figure 3:**
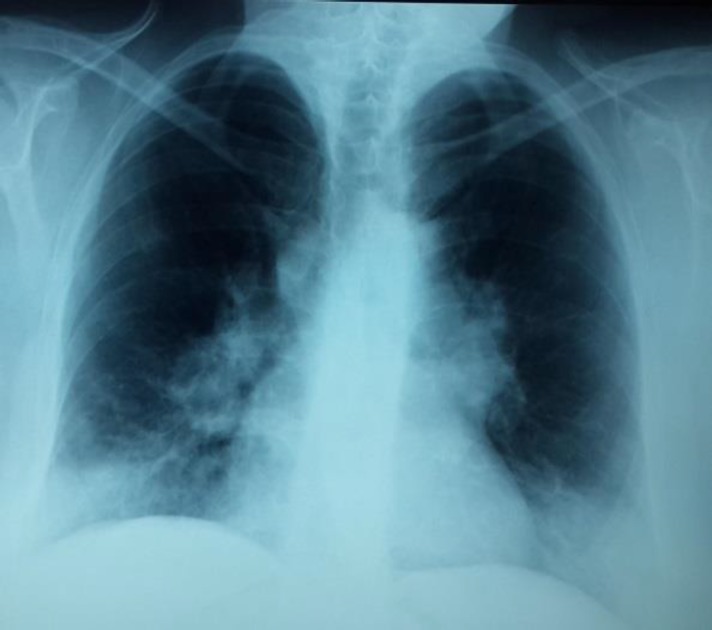
Chest x-rays showed mediastinal enlargement and a reticulonodular infiltrate in the middle and lower lungs

Chest X-ray showed a mediastinal enlargement and a reticulonodular infiltrate in the middle and lower lungs (figure 3).

The chest CT scan confirmed the presence of several and bilateral hilar lymph nodes and parenchymal nodes. The pulmonary functional tests showed a restrictive syndrome. Since sarcoidosis was suspected, bronchoscopy and biopsies were performed showing non-calcified granulomatous reaction without cell necrosis. Ophthalmologist examination showed bilateral sequela of anterior uveitis. 

The patient cumulated 9 points of Amor criteria confirming the diagnosis of SA. A co-occurrence of axial and peripheral SA and sarcoidosis was established. The patient was treated with 15 mg per week of methotrexate and 1mg/kg/day of prednisone for pulmonary disease with good outcomes.

## Discussion

Up to this date, the co-occurrence of sarcoidosis and SA was reported in 17 cases. The originality of our case was the discovery in mean time of a typical SA with ankylosed sacroiliac joints and bamboo column and typical hilar lymph nodes of sarcoidosis.

In previous cases reported, female outnumbered male (sex ratio: 4.1), with age range varying from 42 to 70 years-old. Sarcoidosis was the first disease diagnosed in 7 cases. Simultaneous diagnosis of both diseases was done in 8 patients as in our case. Ankle involvement was the most reported articular manifestation. One case of advanced SA with a bamboo column as in our patient was reported (5). As a complication of both diseases, anterior uveitis was diagnosed in one case (6).

Sacroiliitis has been rarely described in sarcoidosis, mainly in case reports.Different diagnosis may be discussed: pyogenic or mycobacterial infection, malignant process or sarcoid infiltration. While the prevalence of SA in general population is 1 to 1.9%, sacroiliac joint involvement was found in 6.6% of 61 sarcoid patients in one study (7) and in 14.3% of 42 sarcoid patients in another study (8). In both studies, the diagnosis of SA was retained for all patients with sacroiliitis based on ASAS or ESSG criteria. Only one patient of the first study was positive for HLA-B27. Also by reviewing the 17 cases of coexisting sarcoidosis and SA, HLA-B27 was positive only in 4 patients. Rareness of association with common HLA marker of SA suggests that different genetic factors may be involved in the coexistence of the two diseases. HLA-DR5 has been found in sarcoid patients. Different genetic bases (HLA class 1 in SA and HLA class 2 in sarcoidosis) suggest that the association was a coincidence rather than common etiopathogenesis. Nevertheless both diseases have some common features like uveitis, interstitial lung disease, sacroiliitis and good response to anti-TNF α therapy. 

In fact, immunomodulatory agents like anti-TNF α have proven their efficiency in sarcoidosis treatment (9). The three drugs evaluated were infliximab in 232 cases, etanercept in 26 cases and adalimumab in 8 cases (10). There was no study with golimumab or certolizumab. The three commonly used anti-TNF drugs were not equivalent. Infliximab has demonstrated improvement in multiorgan involvement of sarcoidosis: lung, eyes, brain, heart, skin and liver (11). Adalimumab has proven also its efficiency in sarcoidosis treatment even in the case of infliximab intolerance (12). However, etanercept has been studied only for lung involvement and has not demonstrated real improvement (13).

Physiopathogenesis of efficiency of anti-TNF therapy has been explained by a humoral immune reaction. After an exogenous stimulus, immune response is initiated by activation of CD4+ T helper cells. The result is secretion of cytokines acting as chemoattractants and recruiting macrophages, neutrophils and other lymphocytes. Activation of these cells releases TNF α which activate in turn different signaling pathways and mediate granuloma formation (14). Thus, inhibition of TNF α should result in less granuloma formation. Monoclonal antibodies (infliximab and adalimumab) will bind quickly with strong affinity and low dissociation constant to soluble and membrane TNF α. On the other hand, soluble receptor (etanercept) binds only to soluble TNF α. This immune complex is less stable and will be rapidly eliminated in few minutes (15). In some particular situation and for unknown reasons, neutralization of peripheral TNF α is responsible of the activation of specific and auto-reactive lymphocyte T and the result is a granuloma formation and a sarcoid-like reaction (16). 

In 10 years time 71 cases of sarcoid induced reaction to anti-TNF therapy have been reported (17). Mainly the occurrence of respiratory signs despite a stable disease conducts physicians to perform chest x-ray or CT-scan. Hilar and mediastinal adenopathy were the most frequent pulmonary findings and the diagnosis of sarcoidosis was confirmed histologically in 90% of cases (18). Withdrawing the TNF treatment was associated to regression of the sarcoid-like reaction and in most cases steroids were used. 

In conclusions we describe a case of co-occurrence of sarcoidosis and evolved SA. The higher rate of sarcoidosis described as a paradoxical effect of anti-TNF α therapy than a coexistence with SA and physiopathological pathways mediate by TNF α are great arguments for coincidence of this association.
